# Creating a Student-centered Learning Environment: Implementation of Problem-based Learning to Teach Microbiology to Undergraduate Medical Students

**DOI:** 10.7759/cureus.2029

**Published:** 2018-01-05

**Authors:** Venkataramana Kandi, Parimala Reddy Basireddy

**Affiliations:** 1 Department of Microbiology, Prathima Institute of Medical Sciences; 2 II Mbbs, Prathima Institute of Medical Sciences

**Keywords:** microbiology, undergraduate medical students, medical education, problem-based learning

## Abstract

Introduction

Medical education involves training necessary to become a physician or a surgeon. This includes various levels of training like undergraduate, internship, and postgraduate training. Medical education can be quite complex, since it involves training in pre-clinical subjects (anatomy, physiology, biochemistry), the para-clinical subjects (microbiology, pathology, pharmacology, and forensic medicine), and a discrete group of clinical subjects that include general medicine, surgery, obstetrics and gynaecology, ear, nose and throat specialization, paediatrics, cardiology, pulmonology, dermatology, ophthalmology, and orthopaedics, and many other clinical specializations and super specialities (cardio-thoracic surgery, neurosurgery, etc.). Training medical students involves both classroom teaching and practical applications. Classroom teaching is usually confined to didactic lectures, where the teacher unilaterally disseminates the information. This kind of teaching was recently noted to be not very effective in producing better quality medical graduates. The present study aims to introduce problem-based learning (PBL) to teach microbiology to undergraduate medical students and evaluate their perception towards such type of learning.

Methods

A total of 159 students were included in the study. An informed and oral consent was obtained from each participant, and the study was approved by the institutional ethical committee. All the students included in the study were grouped into 14 groups of 11-13 students. Students were carefully grouped ensuring that each group had a good mix that included different levels of achievers. Students were given a detailed introduction to the exercise before they started it. A questionnaire that consisted of 11 points was given to the students and they were asked to give feedback (strongly disagree, disagree, agree to some extent, agree, strongly agree) both on the functioning of PBL and the tutor performance during PBL.

Results

The study included a total of 159 students. Among the study participants, 55 (35%) were male and 104 (65%) were female. There was a positive response towards PBL being instrumental in improving cognitive skills as evidenced by the results (females (59%) and males (29%)) (p=0.191). We found that 61% females and 30% males felt that PBL was the best learning technique, as compared to traditional teaching (p=0.241). Most students were happy with the number of students in a group (females (63%) and males (34%)), but a few students felt that there would have been an improvement in the learning process if the groups were smaller (<10 students) (p=0.239). A positive response was given by the students regarding the feedback encouragement provided by the tutor (females (43%) and males (27%)) (p=0.253). Tutor evaluation by the students revealed some interesting observations, which include an agreement by most students that the tutor had completely avoided traditional teaching (females (55%), males (32%)) during the PBL sessions (p=0.001).

Conclusion

Most students liked PBL as it encouraged group discussions and presentations, which helped in retaining information and improving cognitive skills.

## Introduction

The process of learning and gaining knowledge is called education. The word education is derived from the Latin word 'educationem', which means to grow/improve knowledge, usually by undergoing training [1]. Education involves teaching, training, discussion, and research. There are various stages/levels of education starting from the kindergarten level to the university level. Education can also be done in different forms that include alternate education (home tutoring, open-classroom schools, all forms of education other than the traditional one); indigenous education (educating in one’s own language and culture); and informal education, which was introduced by the United Nations (UN), and called so by the Organization for Economic Co-operation and Development (OECD), which has 35 member countries and encourages out-of-school learning, learning through community centres and youth programmes, and learning by life experiences) [2]. Another form of education is self-education, or self-directed learning (SDL) (Greek: auto-didactism). SDL does not require the guidance of masters; it is independent learning and is not a formal type of education. The most recent and modern method of education is e-learning/open-education, which uses electronic technology. In this method, the students are not required to attend regular classrooms, and most of the training and evaluation happens to be online. Recently, a hybrid of e-learning and traditional training has been used to improve the learning process [3].

Problem-based learning (PBL), considered the most innovative pedagogic technique, was first introduced to teach medicine in the 1950s. PBL is a student-centric learning procedure where a teacher only acts as a moderator. PBL uses a problem as a starting point for learning. In PBL, students learn everything in the context of a medical problem and can solve the problem, as is done in the case of a real patient scenario [4].

Problem-based learning (PBL) practically means learning by experience and is usually self-guided. It is even better if the learning is guided by an expert. PBL can be correlated with the methods applied by ancient humans who had to learn to live through hardships and gradually acquire knowledge, which was used to improve the quality of life by self-experiences. The idea of problem-based learning was implemented during the 1960s to teach medicine at McMaster University, Canada [5].

The main reason for the incorporation of PBL in medical teaching was to ensure that interest was created in the students by preparing them to solve a real-life problem. PBL turns the education system from a teaching paradigm to a learning paradigm, where the traditional lecture is replaced by a real-life problem. Teachers, instead of delivering a routine lecture, only assist/guide the learners who do a self-directed learning, which makes PBL an exciting experience for the learners [6].

The PBL curriculum design involves the development of ill-structured but real-life problems, which work as triggers for learning. The triggers could include videos, tape recordings, placards, labels, pictures, cartoons, postures, and many others, which can initiate the most important aspect of PBL—brainstorming. The next significant step in the PBL process includes independent study (under the guidance of an expert/tutor), where the learners, both individually as well as in groups, acquire information regarding the problem (libraries, books, online, other sources recommended by the guide/expert/tutor). Later in the PBL exercise, the learners, both individually and as a group, present a probable solution to the problem, share the knowledge, carry out group discussions, and present seminars [7].

The aim of this study was to incorporate PBL in the microbiology teaching curriculum and assess the attitude of undergraduate medical students towards PBL and its influence on the microbiology learning process.

## Materials and methods

A total of 159 students were included in the study. An informed oral consent was obtained from each participant and the study was approved by the institutional ethical committee. All the students included in the study were grouped into 14 groups of 11-13 students. The students were carefully grouped ensuring that each group had a good mix of different levels of achievers. The students were given a detailed introduction of the exercise prior to the start of the exercise.

This is a pilot study, the first ever, on implementing PBL in the subject of microbiology. The department of microbiology identified five important microorganisms and infectious diseases frequently causing human infections. *Staphylococcus aureus*, *Streptococcus pyogenes*, *Streptococcus pneumoniae*, *Bacillus anthracis*, *Clostridium *spp were included in the PBL sessions.

The PBL exercise was conducted once a week parallelly with traditional classroom teaching. The department of microbiology, under the leadership of the head of the department, was involved in framing the structure and organization of the PBL exercises, the content to be provided, and creating problem questions for the PBL tutorial. A clear understanding of learning resources and their utilization, group work, attitudes, and communication skills were previously introduced to both the students and the teacher.

Implementation of a PBL exercise

Group Organization

Tutorial groups of 11-13 students, each consisting of a chair, a scribe (usually a student), and a tutor were organized. The chair was responsible for the content and consequence of the PBL exercise, ensuring that the questions were prepared, the students were stimulated, appropriate questions were asked, and the time limit was kept. A scribe made sure that the group was focused, the discussion went in the right direction and summarized and recorded the whole process. Group members actively participated in the discussion, both by listening and speaking. A tutor facilitated the whole process by serving as a resource and evaluating the performance.

Functioning of PBL

The chair of the PBL session described a phenomenon, which was prepared by the staff, to the participants and directed learning activities that revolved around the problem. Later, the groups started a discussion by the activation of prior knowledge of the problem and gathered necessary ideas (learning resources, integration of knowledge from different disciplines, exchange of information) required to learn to solve the problem.

Evaluation of a PBL Exercise

In this exercise, both the student group and the tutor participated to assess the learning experience of an individual student and the group. Areas for improvement were identified after taking suggestions from both the participating members and the chair.

Assessment

This study evaluated the PBL sessions by student feedback (self-evaluation), group evaluation, peer evaluation, teacher feedback, and various forms of presentations as chosen by the student (PowerPoint presentation, summary talks, etc).

After a thorough literature search, a questionnaire was prepared, which consisted of 11 points on which the students were asked to give a feedback (strongly disagree, disagree, agree to some extent, agree, strongly agree) both on the functioning of PBL and the tutor performance during PBL.

Statistical Applications

Microsoft Word and Microsoft Excel were used to perform statistical analysis and draw tables and graphs. The chi-square test for independence was used to determine the relationship between variables of a sample. The following statistical software programs were used for the analysis of the data: SAS 9.2 (Cary, North Carolina, USA), SPSS 15.0 (SPSS Inc., Chicago, IL, USA), Stata 10.1 (StataCorp LP, Texas, USA), MedCalc 9.0.1 (Medcalc software, Ostend, Belgium), Systat 12.0 (Systat Software, Inc., Chicago, IL, USA), and R environment ver.2.11.1 (The R foundation, Vienna, Austria).

## Results

**Figure 1:**
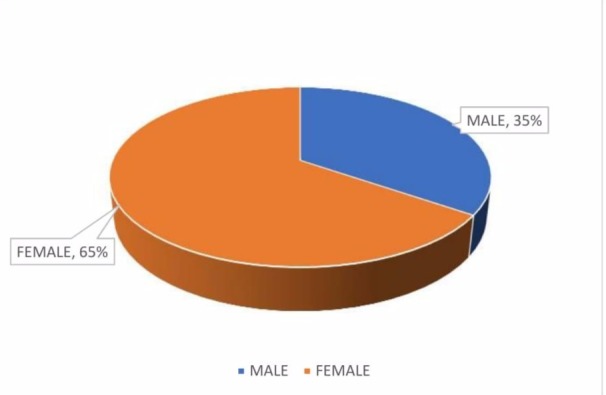
Sex distribution of the study participants

The study included a total of 159 students. Among the study participants, 55 (35%) were male students and 104 (65%) were female students as shown in Figure 1.

The students were asked to respond to a questionnaire consisting of 11 points on a Likert scale (strongly disagree, disagree, agree to some extent, agree, strongly agree). Their feedback on the PBL sessions is shown in Table 1.

**Table 1:** Student feedback on the PBL sessions PBL: Problem based learning

Student feedback on the PBL sessions	Strongly disagree		Disagree		Agree to some extent		Agree		Strongly agree	
	Male (n=55)	Female (n=104)	Male (n=55)	Female (n=104)	Male (n=55)	Female (n=104)	Male (n=55)	Female (n=104)	Male (n=55)	Female (n=104)
PBL sessions helped me to learn more about a topic than a regular lecture	3 (5.5%)	6 (5.8%)	14 (25.5%)	27 (26%)	16 (29.1%)	24 (23.1%)	13 (23.6%)	40 (38.5%)	9 (16.4%)	7 (6.7%)
I liked PBL since group discussions and presentations improved my ability to speak and communicate	2 (3.6%)	4 (3.8%)	7 (12.7%)	6 (5.8%)	11 (20%)	19 (18.3%)	28 (50.9%)	57 (54.8%)	7 (12.7%)	18 (17.3%)
PBL improved my cognitive skills	2 (3.6%)	3 (2.9%)	7 (12.7%)	7 (6.7%)	12 (21.8%)	19 (18.3%)	27 (49.1%)	53 (51%)	7 (12.7%)	22 (21.2%)
PBL is the best method to learn effectively	4 (7.3%)	2 (1.9%)	5 (9.1%)	5 (4.8%)	12 (21.8%)	29 (27.9%)	30 (54.5%)	54 (51.9%)	4 (7.3%)	14 (13.5%)
PBL has no effect on my learning process	3 (5.5%)	0 (0%)	5 (9.1%)	20 (19.2%)	12 (21.8%)	39 (37.5%)	26 (47.3%)	33 (31.7%)	9 (16.4%)	12 (11.5%)
Post PBL presentation further improved knowledge on the subject	1 (1.8%)	1 (1%)	2 (3.6%)	2 (1.9%)	11 (20%)	20 (19.2%)	25 (45.5%)	53 (51%)	16 (29.1%)	28 (26.9%)
I want PBL sessions alone	2 (3.6%)	1 (1%)	10 (18.2%)	4 (3.8%)	18 (32.7%)	36 (34.6%)	21 (38.2%)	55 (52.9%)	4 (7.3%)	8 (7.7%)
I want a hybrid of PBL and traditional teaching methods	3 (5.5%)	0 (0%)	10 (18.2%)	12 (11.5%)	19 (34.5%)	39 (37.5%)	19 (34.5%)	53 (51%)	4 (7.3%)	10 (9.6%)
I want only traditional teaching	3 (5.5%)	2 (1.9%)	3 (5.5%)	3 (2.9%)	13 (23.6%)	21 (20.2%)	21 (38.2%)	57 (54.8%)	15 (27.3%)	21 (20.2%)
Did you feel satisfied with the intergroup interactions?	4 (7.3%)	5 (4.8%)	7 (12.7%)	17 (16.3%)	16 (29.1%)	50 (48.1%)	22 (40%)	27 (26%)	6 (10.9%)	5 (4.8%)
Were you happy with the number of students in a group?	0 (0%)	2 (1.9%)	1 (1.8%)	2 (1.9%)	10 (18.2%)	12 (11.5%)	27 (49.1%)	46 (44.2%)	17 (30.9%)	42 (40.4%)

 Table 2 shows the feedback of the students on the tutors who conducted the PBL sessions.

**Table 2:** Student feedback on the tutors who conducted the PBL sessions PBL: Problem based learning

Student feedback on the tutors who conducted the PBL sessions	Strongly disagree		Disagree		Agree to some extent		Agree		Strongly agree	
	Male (n=55)	Female (n=104)	Male (n=55)	Female (n=104)	Male (n=55)	Female (n=104)	Male (n=55)	Female (n=104)	Male (n=55)	Female (n=104)
Tutor avoided traditional lecturing during PBL sessions	4 (7.3%)	5 (4.8%)	1 (1.8%)	11 (10.6%)	17 (30.9%)	48 (46.2%)	21 (38.2%)	35 (33.7%)	12 (21.8%)	5 (4.8%)
Constant vigil and observation was done by tutor during PBL sessions	0 (0%)	3 (2.9%)	6 (10.9%)	16 (15.4%)	12 (21.8%)	30 (28.8%)	24 (43.6%)	37 (35.6%)	13 (23.6%)	17 (16.3%)
Were you provided with necessary information before the PBL sessions started?	1 (1.8%)	4 (3.8%)	6 (10.9%)	10 (9.6%)	15 (27.3%)	42 (40.4%)	25 (45.5%)	34 (32.7%)	8 (14.5%)	13 (12.5%)
Provided you necessary guidance regarding learning material	3 (5.5%)	3 (2.9%)	4 (7.3%)	23 (22.1%)	19 (34.5%)	44 (42.3%)	23 (41.8%)	26 (25%)	6 (10.9%)	8 (7.7%)
Were the PBL sessions well planned?	9 (16.4%)	5 (4.8%)	28 (50.9%)	52 (50%)	6 (10.9%)	25 (24%)	11 (20%)	19 (18.3%)	1 (1.8%)	3 (2.9%)
Tutor was successful in highlighting the learning objectives	1 (1.8%)	2 (1.9%)	5 (9.1%)	9 (8.7%)	15 (27.3%)	38 (36.5%)	24 (43.6%)	46 (44.2%)	10 (18.2%)	9 (8.7%)
Tutor could make you solve learning issues	14 (25.5%)	17 (16.3%)	29 (52.7%)	59 (56.7%)	6 (10.9%)	15 (14.4%)	4 (7.3%)	10 (9.6%)	2 (3.6%)	3 (2.9%)
Tutor could stimulate you into brainstorming	0 (0%)	4 (3.8%)	3 (5.5%)	9 (8.7%)	17 (30.9%)	22 (21.2%)	25 (45.5%)	46 (44.2%)	10 (18.2%)	23 (22.1%)
Your teachers were attentive during PBL sessions	12 (21.8%)	13 (12.5%)	24 (43.6%)	54 (51.9%)	9 (16.4%)	16 (15.4%)	6 (10.9%)	17 (16.3%)	4 (7.3%)	4 (3.8%)
Were the PBL sessions a great success?	3 (5.5%)	6 (5.8%)	5 (9.1%)	11 (10.6%)	13 (23.6%)	37 (35.6%)	25 (45.5%)	42 (40.4%)	9 (16.4%)	8 (7.7%)
Tutor encourages feedback	5 (9.1%)	15 (14.4%)	6 (10.9%)	22 (21.2%)	10 (18.2%)	22 (21.2%)	26 (47.3%)	36 (34.6%)	8 (14.5%)	9 (8.7%)

The responses of the female students regarding the PBL sessions and their significance in relation to learning microbiology is summarized in Table 3.

**Table 3:** Student feedback on the PBL sessions (female group) PBL: Problem based learning. p value: Probability value. + Suggestive significance (p value: 0.05 < p < 0.10). * Moderately significant (p value: 0.01 < p ≤ 0.05). ** Highly significant (p value: p ≤ 0.01).

Student feedback on the PBL sessions	Strongly disagree	p value	Disagree	p value	Agree to some extent	p value	Agree	p value	Strongly agree	p value
	Female (n=104)		Female (n=104)		Female (n=104)		Female (n=104)		Female (n=104)	
PBL sessions helped me learn more about a topic than a regular lecture	6 (5.8%)	0.935	27 (26%)	0.945	24 (23.1%)	0.406	40 (38.5%)	0.059+	7 (6.7%)	0.055+
I liked PBL since group discussions and presentations improved my ability to speak and communicate	4 (3.8%)	0.947	6 (5.8%)	0.128	19 (18.3%)	0.791	57 (54.8%)	0.639	18 (17.3%)	0.450
PBL improved my cognitive skills	3 (2.9%)	0.796	7 (6.7%)	0.204	19 (18.3%)	0.591	53 (51%)	0.822	22 (21.2%)	0.191
PBL is the best method to learn effectively	2 (1.9%)	0.092+	5 (4.8%)	0.290	29 (27.9%)	0.406	54 (51.9%)	0.753	14 (13.5%)	0.241
PBL has no effect on my learning process	0 (0%)	0.016*	20 (19.2%)	0.095+	39 (37.5%)	0.044+	33 (31.7%)	0.054+	12 (11.5%)	0.393
Post PBL presentation further improved knowledge on the subject	1 (1%)	0.645	2 (1.9%)	0.512	20 (19.2%)	0.907	53 (51%)	0.509	28 (26.9%)	0.771
I want PBL sessions alone	1 (1%)	0.238	4 (3.8%)	0.002**	36 (34.6%)	0.811	55 (52.9%)	0.077+	8 (7.7%)	0.924
I want a hybrid of PBL and traditional teaching methods	0 (0%)	0.016*	12 (11.5%)	0.248	39 (37.5%)	0.713	53 (51%)	0.048*	10 (9.6%)	0.62
I want only traditional teaching	2 (1.9%)	0.225	3 (2.9%)	0.419	21 (20.2%)	0.614	57 (54.8%)	0.046*	21 (20.2%)	0.31
Were you satisfied with the intergroup interactions?	5 (4.8%)	0.522	17 (16.3%)	0.544	50 (48.1%)	0.021*	27 (26%)	0.068+	5 (4.8%)	0.149
Were you happy with the number of students in a group?	2 (1.9%)	0.301	2 (1.9%)	0.963	12 (11.5%)	0.248	46 (44.2%)	0.559	42 (40.4%)	0.239

The responses of the male students regarding the PBL sessions and their significance in relation to learning microbiology is summarized in Table 4.

**Table 4:** Student feedback on the PBL sessions (male group) PBL: Problem based learning p value: Probability value. Suggestive significance (p value: 0.05 < p < 0.10). * Moderately significant (p value: 0.01 < p ≤ 0.05). ** Highly significant (p value: p ≤ 0.01).

Student feedback on the PBL sessions	Strongly disagree	p value	Disagree	p value	Agree to some extent	p value	Agree	p value	Strongly agree	p value
	Male (n=55)		Male (n=55)		Male (n=55)		Male (n=55)		Male (n=55)	
PBL sessions helped me learn more about a topic than a regular lecture	3 (5.5%)	0.935	14 (25.5%)	0.945	16 (29.1%)	0.406	13 (23.6%)	0.059+	9 (16.4%)	0.055+
I liked PBL since group discussions and presentations improved my ability to speak and communicate	2 (3.6%)	0.947	7 (12.7%)	0.128	11 (20%)	0.791	28 (50.9%)	0.639	7 (12.7%)	0.450
PBL improved my cognitive skills	2 (3.6%)	0.796	7 (12.7%)	0.204	12 (21.8%)	0.591	27 (49.1%)	0.822	7 (12.7%)	0.191
PBL is the best method to learn effectively	4 (7.3%)	0.092+	5 (9.1%)	0.290	12 (21.8%)	0.406	30 (54.5%)	0.753	4 (7.3%)	0.241
PBL has no effect on my learning process	3 (5.5%)	0.016*	5 (9.1%)	0.095+	12 (21.8%)	0.044+	26 (47.3%)	0.054+	9 (16.4%)	0.393
Post PBL presentation further improved knowledge on the subject	1 (1.8%)	0.645	2 (3.6%)	0.512	11 (20%)	0.907	25 (45.5%)	0.509	16 (29.1%)	0.771
I want PBL sessions alone	2 (3.6%)	0.238	10 (18.2%)	0.002**	18 (32.7%)	0.811	21 (38.2%)	0.077+	4 (7.3%)	0.924
I want a hybrid of PBL and traditional teaching methods	3 (5.5%)	0.016*	10 (18.2%)	0.248	19 (34.5%)	0.713	19 (34.5%)	0.048*	4 (7.3%)	0.62
I want only traditional teaching	3 (5.5%)	0.225	3 (5.5%)	0.419	13 (23.6%)	0.614	21 (38.2%)	0.046*	15 (27.3%)	0.31
Were you satisfied with the intergroup interactions?	4 (7.3%)	0.522	7 (12.7%)	0.544	16 (29.1%)	0.021*	22 (40%)	0.068+	6 (10.9%)	0.149
Were you happy with the number of students in a group?	0 (0%)	0.301	1 (1.8%)	0.963	10 (18.2%)	0.248	27 (49.1%)	0.559	17 (30.9%)	0.239

The feedback of the female students on the tutors who conducted the PBL sessions is summarized in Table 5.

**Table 5:** Student feedback on the tutors who conducted the PBL sessions (female group) PBL: Problem based learning. p value: Probability value. + Suggestive significance (p value: 0.05 < p < 0.10). * Moderately significant (p value: 0.01 < p ≤ 0.05). ** Highly significant (p value: p ≤ 0.01).

Student feedback on the tutors who conducted the PBL sessions	Strongly disagree	p value	Disagree	p value	Agree to some extent	p value	Agree	p value	Strongly agree	p value
	Female (n=104)		Female (n=104)		Female (n=104)		Female (n=104)		Female (n=104)	
Tutor avoided traditional lecturing during PBL sessions	5 (4.8%)	0.522	11 (10.6%)	0.047*	48 (46.2%)	0.063+	35 (33.7%)	0.570	5 (4.8%)	0.001**
Constant vigil and observation was done by tutor during PBL sessions	3 (2.9%)	0.204	16 (15.4%)	0.437	30 (28.8%)	0.339	37 (35.6%)	0.320	17 (16.3%)	0.264
Were you provided with necessary information before the PBL sessions started?	4 (3.8%)	0.486	10 (9.6%)	0.796	42 (40.4%)	0.101	34 (32.7%)	0.113	13 (12.5%)	0.717
Provided you necessary guidance regarding learning material	3 (2.9%)	0.419	23 (22.1%)	0.018*	44 (42.3%)	0.341	26 (25%)	0.029*	8 (7.7%)	0.496
Were the PBL sessions well planned?	5 (4.8%)	0.014*	52 (50%)	0.913	25 (24%)	0.047*	19 (18.3%)	0.791	3 (2.9%)	0.683
Tutor was successful in highlighting the learning objectives	2 (1.9%)	0.963	9 (8.7%)	0.926	38 (36.5%)	0.237	46 (44.2%)	0.943	9 (8.7%)	0.078+
Tutor could make you solve learning issues	17 (16.3%)	0.168	59 (56.7%)	0.629	15 (14.4%)	0.534	10 (9.6%)	0.620	3 (2.9%)	0.796
Tutor could stimulate you into brainstorming	4 (3.8%)	0.141	9 (8.7%)	0.468	22 (21.2%)	0.174	46 (44.2%)	0.883	23 (22.1%)	0.561
Your teachers were attentive during PBL sessions	13 (12.5%)	0.125	54 (51.9%)	0.32	16 (15.4%)	0.872	17 (16.3%)	0.354	4 (3.8%)	0.347
Were the PBL sessions a great success?	6 (5.8%)	0.935	11 (10.6%)	0.767	37 (35.6%)	0.123	42 (40.4%)	0.538	8 (7.7%)	0.092+
Tutor encouraged feedback	15 (14.4%)	0.335	22 (21.2%)	0.060+	22 (21.2%)	0.657	36 (34.6%)	0.120	9 (8.7%)	0.253

Table 6 shows the feedback of the male students on the tutors who conducted the PBL sessions.

**Table 6:** Student feedback on the tutors who conducted the PBL sessions (male group) PBL: Problem based learning. p value: Probability value. + Suggestive significance (p value: 0.05 < p < 0.10). * Moderately significant (p value: 0.01 < p ≤ 0.05). ** Highly significant (p value: p ≤ 0.01).

Student feedback on the tutors who conducted the PBL sessions	Strongly disagree	p value	Disagree	p value	Agree to some extent	p value	Agree	p value	Strongly agree	p value
	Male (n=55)		Male (n=55)		Male (n=55)		Male (n=55)		Male (n=55)	
Tutor avoided traditional lecturing during PBL sessions	4 (7.3%)	0.522	1 (1.8%)	0.047*	17 (30.9%)	0.063+	21 (38.2%)	0.570	12 (21.8%)	0.001**
Constant vigil and observation was done by tutor during PBL sessions	0 (0%)	0.204	6 (10.9%)	0.437	12 (21.8%)	0.339	24 (43.6%)	0.320	13 (23.6%)	0.264
Were you provided with necessary information before the PBL sessions started?	1 (1.8%)	0.486	6 (10.9%)	0.796	15 (27.3%)	0.101	25 (45.5%)	0.113	8 (14.5%)	0.717
Provided you necessary guidance regarding learning material	3 (5.5%)	0.419	4 (7.3%)	0.018*	19 (34.5%)	0.341	23 (41.8%)	0.029*	6 (10.9%)	0.496
Were the PBL sessions well planned?	9 (16.4%)	0.014*	28 (50.9%)	0.913	6 (10.9%)	0.047*	11 (20%)	0.791	1 (1.8%)	0.683
Tutor was successful in highlighting the learning objectives	1 (1.8%)	0.963	5 (9.1%)	0.926	15 (27.3%)	0.237	24 (43.6%)	0.943	10 (18.2%)	0.078+
Tutor could make you solve learning issues	14 (25.5%)	0.168	29 (52.7%)	0.629	6 (10.9%)	0.534	4 (7.3%)	0.620	2 (3.6%)	0.796
Tutor could stimulate you into brainstorming	0 (0%)	0.141	3 (5.5%)	0.468	17 (30.9%)	0.174	25 (45.5%)	0.883	10 (18.2%)	0.561
Your teachers were attentive during PBL sessions	12 (21.8%)	0.125	24 (43.6%)	0.32	9 (16.4%)	0.872	6 (10.9%)	0.354	4 (7.3%)	0.347
Were the PBL sessions a great success?	3 (5.5%)	0.935	5 (9.1%)	0.767	13 (23.6%)	0.123	25 (45.5%)	0.538	9 (16.4%)	0.092+
Tutor encouraged feedback	5 (9.1%)	0.335	6 (10.9%)	0.060+	10 (18.2%)	0.657	26 (47.3%)	0.120	8 (14.5%)	0.253

## Discussion

The academic curriculum of MBBS (Bachelor of Medicine and Bachelor of Surgery) students requires them to learn four subjects in the second year, which include microbiology, pathology, pharmacology, and forensic medicine. Students are also required to attend clinical postings, while simultaneously learning the clinical subjects. They are also required to visit rural health centres to gain awareness of and expertise in public health concerns in that region. Microbiology is a vast subject that deals with the study of various microorganisms, the diseases they cause, their pathogenicity, epidemiology, and the laboratory diagnosis. It is very important for a teacher to know how much the learners understand what is being taught in a classroom. In traditional classroom teaching, students are taught about most microorganisms that cause human diseases. However, there are more than 50 important microbial infections, and students usually experience difficulty in remembering all of them and often confuse one microbe with the other.

In traditional teaching, a medical teacher unilaterally gives lectures containing information about the microorganisms. Often, students really do not know which aspects of the microorganisms are important from the patient/practice perspective. This issue can be solved by incorporating problem-based learning (PBL) in the academic curriculum. PBL has not yet been implemented to teach microbiology for MBBS students in India. Previously, a study by Ciraj AM et al. from Manipal incorporated case-based learning to teach microbiology to MBBS students. This study noted that PBL was able to enhance the students' learning abilities and that there was improvement in the analytic, collaborative, and communication skills of students [8].

The perception of students towards PBL could be indifferent as most medical schools follow traditional teaching. Incorporation of PBL as a curriculum must be done cautiously, and there is a need for assessing students' response towards acceptance of PBL.

Our study shows that 44% of female students and 24% of male students responded positively and experienced that PBL sessions helped them learn more about a topic than a regular lecture did (p=0.055). Students (females (59%) and males (29%)) liked the PBL sessions and experienced that group discussions and presentations of PBL were instrumental in improving their speaking and communicating skills (p=0.450). There was a positive response towards PBL being instrumental in improving cognitive skills as evidenced by the results (females (59%) and males (29%)) (p=0.191).

We found that 61% females and 30% males felt that PBL was the best learning technique as compared to traditional teaching (p=0.241). The study found that 53% of females and 29% of males noted that PBL had no effect on their learning process (p=0.393). This is an interesting and contrasting opinion, as the study participants gave a positive response to previous questions.

We found that 63% female and 26% male students opined that they want PBL alone as a teaching method as compared to traditional teaching (p=0.924). A hybrid curriculum having both PBL as well as traditional teaching was opted by 60% females and 25% male students (p=0.62). Only 49% of female students and 31% of male students felt that they only require traditional teaching (p=0.31). There was a positive response regarding intergroup interactions during the PBL sessions (females (51%) and males (28%)) (p=0.149).

Most students were happy with the number of students in a group (females (63%) and males (34%)), but a few students felt that there could an improvement in the learning process if the groups were smaller (<10 students) (p=0.239).

Tutor evaluation by the students revealed some interesting observations, which include an agreement by most students that the tutor had completely avoided traditional teaching (females (55%) and males (32%)) during the PBL sessions (p=0.001) and that the tutor was on a constant vigil during a PBL session (females (53%) and males (31%)) (p=0.264). A positive tutor feedback was also given by the students regarding the necessary information provided before starting (pre-PBL sensitization) (females (56%) and males (30%)) (p=0.717). The students also felt that the tutor was successful in providing necessary guidance regarding learning issues (females (49%) and males (30%)) (p=0.496).

There was negative response from the students regarding the planning of PBL sessions (females (36%) and males (23%)) (p=0.683). Most students felt that the tutor was successful in highlighting the learning objectives (females (59%) and males (30%)) (p=0.078) and had stimulated brainstorming (females (57%) and males (33%)) (p=0.561).

In contrast, the students responded negatively about the role played by the tutor in helping students solve their learning issues (females (48%) and males (27%)) (p=0.796). Most students also felt that the tutor was not attentive during a PBL session (females (42%) and males (23%)) (p=0.347). This could be attributed to the increased number of PBL groups as compared to the number of tutors and the fact that each tutor had to handle more than one group.

A positive response was given by the students regarding the feedback encouragement provided by the tutor (females (43%) and males (27%)) (p=0.253).

In spite of all the contrasting opinions by the students to different aspects of the PBL, the overall response regarding the PBL sessions was encouragingly positive (females (55%) and males (32%)) (p=0.092).

PBL and its application in teaching microbiology for medical students has not been adequately studied. The PBL curriculum was applied to laboratory medicine residents in France. This study done by Lepiller Q et al. noted that PBL could be instrumental in motivating students and boosting their interest towards learning virology [9].

A study from India by Ciraj AM et al., noted that case-based learning (CBL) was efficient in enhancing the learning abilities in microbiology among medical students. This study observed that there was an improvement in the analytic, collaborative, and communication skills of students [8].

Another observation from India by Saha R et al. noted that it is important to take the opinion of the students/participants/learners before implementing newer teaching methodologies. Mere acceptance of newer techniques does not guarantee the desired results if the students' perceptions are not taken into consideration [10].

CBL was evaluated for teaching medical microbiology by Blewett EL et al. at the Oklahoma State University Center for Health Sciences (OSU-CHS), Oklahoma, USA. The results of this study noted that CBL was instrumental in improving the case solving abilities of the learners and recommended further prospective studies to assess its overall importance in learning outcomes [11]. A recent report from India by Dubey et al., who evaluated the importance of case-based learning to teach pathology to medical students, noted that there was an improvement in cognitive skills among the students who participated in such type of learning [12].

A large scale systematic review and meta-analysis was performed to assess the usefulness of PBL in nursing education during the period 1960-2012 by Kong L-N et al. This study noted that there was an improvement in the critical thinking of nursing students post PBL sessions [13].

A recent research by Holen A et al. has evaluated the perceptions of medical students in various levels of their course and correlated them with the sociocultural aspects of the students. This study included students from Norway, North Dakota, and Nepal. Results of this study revealed that the preference towards a PBL curriculum could be influenced by the social and cultural background of the participants and their perceptions cannot be the same at various stages of the course [14].

PBL was implemented and evaluated for its efficacy in long-term retention of physiology among medical students in Iran. This study included 39 medical students and was conducted at Kerman University of Medical Sciences, Kerman, Iran between 2006 and 2010. Participants were divided into three groups of 13 students each. The results of this study observed that the PBL groups performed better in the exams and were able to retain the subject for a longer period. However, the final exam results were not found to be significant in terms of PBL and non-PBL instructed students [15]. A study by Schmidt HG et al. from Netherlands noted that the PBL curriculum improves the learning process due to both individual as well as group learning activities. PBL also activates the student’s prior knowledge on the subject and improves decision making [16].

A study from the United Kingdom by Tsigarides J et al. concluded that the PBL curriculum has not been implemented throughout the world although it evolved more than 50 years back. This study also had observed that PBL cannot influence the future subject choice of medical students [17]. Harasym PH et al. in their report suggested that PBL stimulates the whole brain as compared to the traditional classroom teaching which can only activate the left side of the brain. This study has also elaborated the disadvantages of PBL in teaching ethical decision skills [18].

As evidenced from the literature, PBL was started in Western countries like the USA and the UK and was later adopted in the Third World countries. A study from Pakistan observed that PBL may be difficult to practice due to the large number of students and their different cultural, lingual, and social backgrounds. PBL sessions might not be possible also because of a lack of adequate infrastructure, including library facilities. Previous learning and educational background also influence the success of a PBL curriculum [19].

## Conclusions

The inclusion of PBL to teach microbiology to undergraduate medical students received a mixed response. Although most students welcomed it, there was confusion among the students mostly because they had never been exposed to this type of learning experience. The students were not happy with the number of students in each group and the timing and planning of PBL sessions, since the academic schedule was already busy with routine teaching and assessment exams. Although the tutors were successful in initiating brainstorming and encouraging feedback, the students felt that the tutor was not able to solve the learning issues of the students and was not attentive during a PBL session. Most students liked PBL as it encouraged group discussions and presentations, which help in retaining the subject and improving cognitive skills. From the results of the current study, we recommend that in situations where students have been previously trained only in a traditional teaching setting, PBL sessions should be planned carefully, preferably by conducting small group sensitization programmes or workshops. Initially, PBL sessions should be confined to a few important infectious diseases.
